# Effects of Resin Chemistries on the Selective Removal of Industrially Relevant Metal Ions Using Wafer-Enhanced Electrodeionization

**DOI:** 10.3390/membranes11010045

**Published:** 2021-01-09

**Authors:** Humeyra B. Ulusoy Erol, Christa N. Hestekin, Jamie A. Hestekin

**Affiliations:** Ralph E. Martin Department of Chemical Engineering, University of Arkansas, Fayetteville, AR 72701, USA; hbulusoy@uark.edu (H.B.U.E.); chesteki@uark.edu (C.N.H.)

**Keywords:** selective separation, ion-exchange resin, wafer-enhanced electrodeionization, desalination

## Abstract

Wafer-enhanced electrodeionization (WE-EDI) is an electrically driven separations technology that occurs under the influence of an applied electric field and heavily depends on ion exchange resin chemistry. Unlike filtration processes, WE-EDI can be used to selectively remove ions even from high concentration systems. Because every excess ion transported increases the operating costs, the selective separation offered by WE-EDI can provide a more energy-efficient and cost-effective process, especially for highly concentrated salt solutions. This work reports the performance comparison of four commonly used cation exchange resins (Amberlite IR120 Na^+^, Amberlite IRP 69, Dowex MAC 3 H^+^, and Amberlite CG 50) and their influence on the current efficiency and selectivity for the removal of cations from a highly concentrated salt stream. The current efficiencies were high for all the resin types studied. Results also revealed that weak cation exchange resins favor the transport of the monovalent ion (Na^+^) while strong cation exchange resins either had no strong preference or preferred to transport the divalent ions (Ca^2+^ and Mg^2+^). Moreover, the strong cation exchange resins in powder form generally performed better in wafers than those in the bead form for the selective removal of divalent ions (selectivity > 1). To further understand the impact of particle size, resins in the bead form were ground into a powder. After grinding the strong cation resins displayed similar behavior (more consistent current efficiency and preference for transporting divalent ions) to the strong cation resins in powder form. This indicates the importance of resin size in the performance of wafers.

## 1. Introduction

The increase in population and industrial development has triggered physical and economic water scarcity. For instance, in various industries such as the semiconductor, pharmaceutical, power, and hydraulic fracturing industries, an average facility can use 2 to 4 million gallons of water per day [1]. Specifically, the consumption of large volumes of fresh water and the generation of highly contaminated wastewater has drawn negative attention from both the public and environmental groups. Besides this attention, excessive freshwater use can create hardships for industries, households, farmers, and wildlife [2]. Hydraulic fracturing, commonly known as fracking, is used to release natural gas and oil and also uses large amounts of water in its production [3,4]. Produced wastewater contains a high concentration of dissolved solids which often exceeds 50,000 parts per million (ppm) and is about 2–6 times higher than seawater concentration [5]. The fracking wastewater contains divalent cations (such as calcium and magnesium) and monovalent ions (such as sodium and potassium) as well as other anions, chemicals, and bacteria [6].

Due to the high concentration of dissolved solids, fracking wastewater can threaten the environment and alter the health of agriculture, aquatic life, and humans. Considering the health threats, fracking water cannot be discharged into freshwater streams or treated at municipal wastewater treatment plants. Currently, there are several ways to dispose of fracking wastewater with the cost ranging from $1 to $10 per barrel [7]. In addition, logistics and water hauling can increase the water management costs when the disposal outlet is not nearby, and it may increase the cost of disposal to $94 per barrel per hour of transport [7]. 

Hence, there is a need for on-site wastewater treatment to minimize the freshwater use and damaging effects of fracking wastewater. If the wastewater can be reused or reduced, then the expenses from transportation and disposal can be decreased or eliminated. Membrane-based technologies have become a remedy for the removal of particulates, ionic, gaseous, and organic impurities from aqueous streams without the use of hazardous chemicals due to their reliability and cost-effectiveness. Wastewater treatment technologies using membranes appear to be the more practical and feasible strategies to overcome one of the primary issues the world faces; the shortage of freshwater supplies and degradation of water quality [8]. Membrane technologies also have essential advantages such as the simplicity of operation, high flexibility and stability [9], low energy requirements [10], high economic compatibility [11], and easy control of operations and scale-up under a broad array of operating conditions and good compatibility between different integrated membrane system operations [12]. 

Electrodeionization (EDI) is a hybrid technology that is based on electrodialysis (ED), which employs electrical current and semi-impermeable membranes, and ion exchange (IE) that contains ion exchange resins [13] to overcome the disadvantages of both technologies such as concentration polarization, chemical regeneration [14], and excessive power utilization at low ion concentrations [15,16,17]. EDI can be operated in both continuous and batch modes and does not require a separate step to regenerate resins. Furthermore, EDI can work with low concentration streams with a lower power requirement compared to ED [15,16,18]. 

Even though there are major advantages of EDI over ED and ion exchange processes, there are also several disadvantages of EDI. The ion exchange resins are inserted into a pair of anionic- and cationic-exchange membranes loosely. This loose resin structure complicates sealing between compartments and leads to leakage of ions from one compartment to another due to convection instead of diffusion [19,20]. Another disadvantage of loose resins in EDI systems is the uneven distribution of flow within the channels which decreases the separation efficiency [20,21,22,23]. Previous studies have found ways to eliminate leakage issues by using spiral-wound configurations [24] or the channeling problem by immobilizing the resin using magnetic fields [25]. Each method was able to eliminate only one of the disadvantages of conventional EDI. Therefore, there was a need for a new system specifically designed to overcome both disadvantages. As a result, an integrated approach, wafer-enhanced electrodeionization (WE-EDI), was proposed by Arora et al. [26]. 

The wafer-enhanced electrodeionization (WE-EDI) is one of the methods that enable on-site wastewater treatments and maintenance, and removal of hardness causing ions and metals [26,27]. In WE-EDI, the loose ion exchange resin structure of conventional EDI is replaced by a wafer inserted between the two membranes as the spacer. The wafer is a mixture of immobilized cation- and anion-exchange resins using a polymer as a binding agent. Compared to conventional EDI, WE-EDI can be easily built and run more efficiently, and it prevents uneven flow distribution and leakage of ions between the compartments simultaneously [28]. Because there is less leakage, WE-EDI can be used for more selective separations such as the removal of acidic impurities from corn stove hydrolysate liquor, CO_2_ capture, and purification of organic acids [26,29]. 

Besides treating wastewater for the removal of impurities, there is a need for an efficient and economical process of ion-selective separation. In wastewater treatment processes, not every ion has the same priority to be removed. Depending on the application, the user may need a selective removal of an ion relative to the remaining ions in the system. Also, because every ion transported that does not need to be transported increases the operating costs, there is a need for ion selectivity to create an energy-efficient and cost-effective process. Ion selectivity in WE-EDI processes heavily depends on ion exchange resin chemistry [23]. However, there are no studies that show the effect of commonly used resins (Amberlite IR 120 Na^+^, Amberlite IRP 69, Amberlite CG 50, and Dowex MAC 3 H^+^) on the ion selectivity and current efficiency in systems with a high salt concentration to the best of our knowledge. Amberlite IR 120 Na^+^ and Amberlite IRP 69 are strong cation exchange resins whereas Amberlite CG 50 and Dowex MAC 3 H^+^ are weak cation exchange resins. These resins are widely used in applications of conventional EDI and ion exchange chromatography such as metal removal [30,31,32], water softening [33,34], drug delivery [35], and enzyme immobilization and purification [36,37]. While these four resins have been commonly used in applications requiring ion transport at low salt concentrations, this study explores their use for selective and energy-efficient removal of ions in a highly concentrated system using wafer-enhanced electrodeionization (WE-EDI). The unique wafers used in WE-EDI enhance the effects of transport by diffusion. Therefore, the effect of resin size in resins with the same chemistry was also evaluated.

## 2. Materials and Methods 

### 2.1. Chemicals

Cationic exchange resins (Amberlite IR 120 Na^+^, Amberlite IRP 69, Dowex MAC 3 H^+^, and Amberlite CG 50), anionic exchange resin (Amberlite IRA-400 Cl^−^), sucrose, low-density polyethylene, sodium chloride, magnesium chloride, and calcium chloride were purchased from VWR International. The technical specifications of each resin are shown in Table 1. Neosepta food-grade anionic and cationic exchange membranes (AMX and CMX, respectively) were purchased from Ameridia Innovative Solutions, Inc. (Somerset, NJ, USA)

### 2.2. Wafer Composition, Fabrication, and System Setup

**Figure 1:**
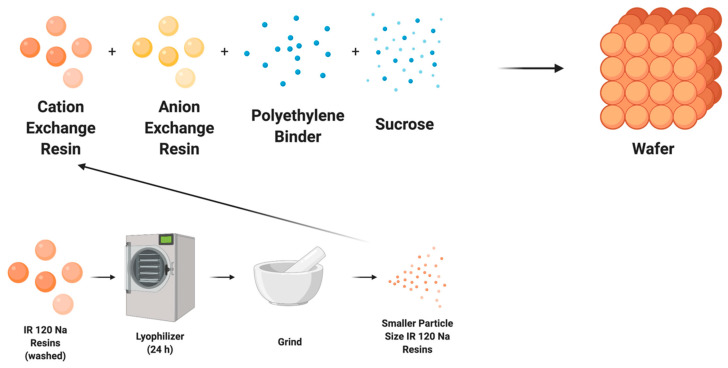
Illustration of typical wafer fabrication and particle size reduction (grinding) of ion exchange resins for wafer fabrication.

The wafer recipe has been previously published [23], but briefly consists of anion and cation exchange resins, polymer, and sucrose (Figure 1). The cationic exchange resins used were Amberlite IR 120 Na^+^, Amberlite IRP 69, Dowex MAC 3 H^+^, and Amberlite CG 50. The first two are strong cationic exchange resins and the latter two are weak cationic exchange resins. The anion exchange resin bead was Amberlite IRA 400 Cl^−^. Polyethylene (500 micron-low density) and sucrose were used to bind the resins and create porosity, respectively. The ratios of cation exchange resin, anionic resin, polymer, and sucrose in the mixture were 23:23:10:15, respectively. The mixture then was uniformly combined using a FlackTeck Inc (Landrum, SC, USA). SpeedMixer™ (model: DAC 150 SP) at a rate of 300 rpm for 5 s. The combined mixture for the wafer was cast in a steel mold and placed in a Carver press (model 3851-0) heated to 250 °F at 10,000 psi for ninety min. This process was followed by a 20-min cooling period via pressurized air treatment. The wafer was pre-soaked in deionized (DI) water for 24-h to create porosity. The thickness of the final product was 2 mm. The wafer was then cut to size to fit within the WE-EDI cell.

Membranes used in the WE-EDI system were Neosepta food-grade AMX and CMX membranes and were conditioned in the dilute (feed) solution (described in the next section) 24 h prior to the experiments. WE-EDI was performed within a Micro Flow Cell (ElectroCell North America, Inc.). The MicroFlow Cell was tightened to 25 in-lbs across all bolts to ensure even flow throughout the system and prevent leakage. The cations tested for selective separation were Na^+^, Ca^2+^, and Mg^2+^ and the counter ion for all cations was Cl^−^. 

### 2.3. Size Reduction for IR 120 Na

To compare the effects of resin size on the system performance, the size of the IR 120 Na^+^ resins was reduced (Figure 1). The IR 120 Na^+^ resins were first washed with deionized water and then dried using a freeze dryer (Labconco FreeZone Plus 12 Liter #7960044, Kansas City, MO, USA). Dried resins were ground using a mortar and pestle and passed through sieves to get resin particles of less than 0.149 mm (100 mesh). Ground resins were then made into a wafer using the same recipe given in Section 2.2.

### 2.4. Particle Image Analysis

Both the original IR 120 Na^+^ and the ground IR 120 Na^+^ resins were examined with an optical microscope. The calibration and particle size detection were completed with ImageJ image processing tool [39].

### 2.5. WE-EDI Chamber Setup and Sample Collection

**Figure 2:**
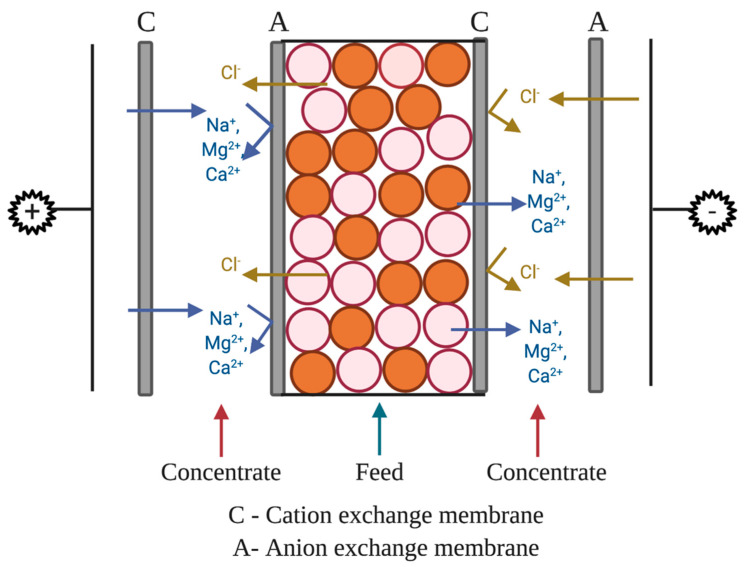
Illustration for wafer-enhanced electrodeionization (EDI) setup.

The setup (Figure 2) for ion removal used four separate solutions of equal volume. The concentrate solution was 300 mL of 2% wt (20 g/L in DI water) sodium chloride solution. The two rinse chamber solutions were 300 mL of 0.3 M (42.6 g/L in DI water) sodium sulfate (Na_2_SO_4_). The feed (dilute) was 50,000 ppm sodium (126.8 g of NaCl/L in DI water), 1000 ppm of calcium (2.7 g of CaCl_2_/L in DI water), and 1000 ppm of magnesium (3.9 g of MgCl_2_/L in DI water). The dilute (feed) stream is the solution from which ions are being diluted (i.e., transported out of or removed).

All experiments were performed in a continuous mode with recycling. A constant current of 0.2 Amps was used for all experiments. Experiments were run for 8 h, with samples collected at the initial (0-h), 2-h, 4-h, and 8-h marks. To determine the concentration of individual ions, ion chromatography (Dionex ™ ICS-6000 Standard Bore and Microbore HPIC ™ Systems, Thermo Scientific, Waltham, MA, USA) was used because of its speed, precision, and sensitivity.

### 2.6. Statistical Analysis

Statistical differences in the data were determined using an unpaired *t*-test in GraphPad QuickCalcs (GraphPad Software Inc., San Diego, CA, USA). Values were considered to have a statistically significant difference if the *p*-value was less than 0.05. 

### 2.7. FTIR-ATR Spectroscopy

The changes to the chemistry of the resin in the wafer were identified using Fourier Transform Infrared—Attenuated Total Reflection (FTIR-ATR) Spectroscopy (Perkin Elmer LR64912C, Waltham, MA, USA). The individual peaks were evaluated in terms of wavenumber and intensity.

## 3. Results and Discussion

### 3.1. Current Efficiency

The current efficiency (*η*) for the WE-EDI system indicates how efficiently a particular ion is being transferred across the membranes and the wafer due to the electrical field applied to the system. It is defined as:

(1) $$\eta =\frac{zFV\left(C_{i}-C_{f}\right)}{tIM_{w}}\times 100\%,$$

where *z* is the ionic valence of the ion (2 for calcium and magnesium, and 1 for sodium), *F* is Faraday’s constant, *V* is the volume of the feed chamber, *C_i_* is the initial concentration of the ion in the feed chamber, *C_f_* is the final concentration of the ion in the feed chamber, *t* is the total operation time, *I* is the current, and *Mw* is the molecular weight of the ion.

**Figure 3:**
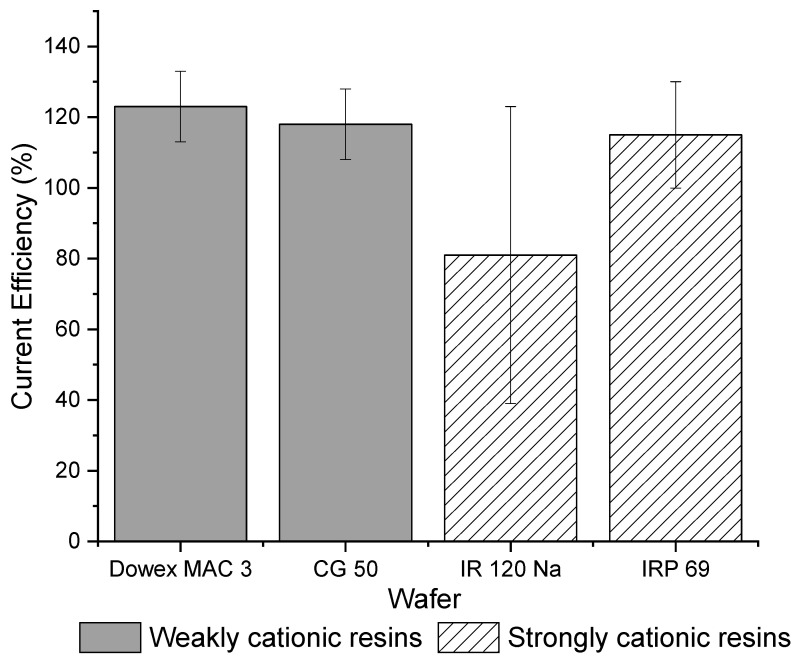
Overall current efficiencies for strong (IR 120 Na^+^ and IRP 69) and weak cation exchange wafers (Dowex MAC 3 H^+^ and CG 50).

Figure 3 shows that the total current efficiency is similar between weak cation exchange and strong cation exchange wafers. The total current efficiency for each strong cation exchange resin wafer was close to 100% and for each weak cation, resin wafer was over 100%. While current efficiencies should be below 100%, other studies have previously reported efficiencies greater than 100%. Pan et al., showed that current efficiency increased in resin wafer EDI as the ion concentration in the dilute stream increased [20]. Luo and Wu [40] observed that the overall current efficiency of their system was greater than 100% at high concentrations. Lopez and Hestekin [29] reported that high ion diffusion during the experiment coupled with ion transport due to potential gradients can cause greater than 100% current efficiency. Another reason why these current efficiencies may exceed 100% is that the concentration of the solution in the dilute chamber is higher than in the concentrate chamber and therefore the electrically driven transport is being assisted by the concentration gradient. In this study, the strong cation exchange IRP 69 resin wafer had a current efficiency that was more consistently approximately 100% whereas the IR 120 Na^+^ wafer showed a lot of variabilities, which makes it less desirable for the selective removal of ions. In terms of the weak cation exchange resin wafers, both resin wafers showed similar average values and smaller variability in their current efficiencies. 

### 3.2. Selectivity

Selectivity is a measure of the removal rate of one ion compared to another. Selectivity was determined using the separation coefficient (*α*) and was calculated using the following equation:

(2) $$\alpha =\frac{\left(C_{i}^{f}-C_{i}^{s}\right)/C_{i}^{s}}{\left(C_{j}^{f}-C_{j}^{s}\right)/C_{j}^{s}},$$

where *C_i_^f^* is the final concentration of ion *i* (calcium or magnesium ion), *C_i_^s^* is the starting concentration of ion *i*, *C_j_^f^* is the final concentration of ion *j* (sodium ion), and *C_j_^s^* is the starting concentration of ion *j.* If *α* is greater than one, it indicates the preferential transport of ion *i*. If *α* is less than one, then it indicates the preferential transport of ion *j*. 

**Figure 4:**
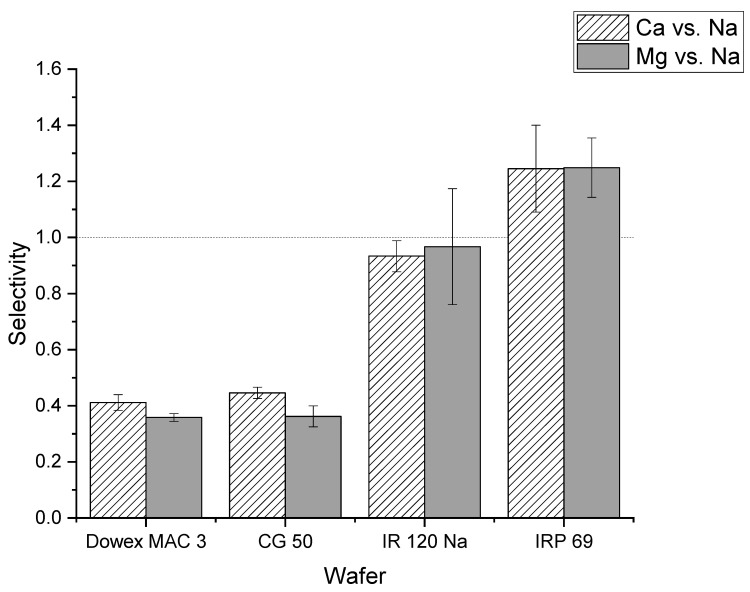
Comparison of selectivity values of calcium and magnesium relative to sodium for different strong cation exchange (IR 120 Na^+^ and IRP 69) and weak cation exchange (Dowex MAC 3 H^+^ and CG 50) resin wafers.

Figure 4 shows the selectivity values for calcium and magnesium relative to sodium for strong and weak cation exchange resin wafers. The selectivity of calcium to sodium was greater than one for the IRP 69 resin wafer (strong cation exchange) which indicated that calcium ions were preferentially transported compared to sodium ions. In the IR 120 Na^+^ resin (strong cation exchange), the selectivity for calcium relative to sodium was close to one which indicated that there was not a strong preference for the transport of sodium or calcium ions. The statistical analysis showed that there was a statistically significant difference between IR 120 Na^+^ and IRP 69 resins for calcium selectivity (*p* < 0.02). In Dowex MAC 3 H^+^ and CG 50 (weak cation exchange resin wafers), the selectivity values for calcium relative to sodium were less than one which indicated that both resin wafers prefer to transport sodium ions over calcium ions. Our statistical analysis showed no difference between Dowex MAC 3 H^+^ and CG 50 resin wafers for calcium removal (*p* > 0.2).

A similar situation was observed for the selectivity of magnesium relative to sodium. The IRP 69 demonstrated a selectivity greater than one, indicating that magnesium was preferentially transported over sodium. For IR 120 Na^+^ resin, the selectivity was at or below one indicating that there was no preference for the transport of magnesium. However, statistical analysis showed that the difference between IR 120 Na^+^ and IRP 69 resin for magnesium selectivity was not significant (*p* > 0.15). In the weak cation exchange resin wafers formed from Dowex MAC 3 H^+^ and CG 50, the selectivity values were less than one which indicated that both resin wafers preferred to transport sodium ions over magnesium ions. The statistical analysis showed no difference between Dowex MAC 3 H^+^ and CG 50 resin wafers for magnesium removal (*p* > 0.8).

It is well established that resins with sulfonic acid groups have a higher affinity for divalent ions than resins with carboxylic acid functional groups [41,42]. For the Amberlite IR 120 Na^+^ sulfonic acid resin, it has been previously reported that the order of selectivity is Ca^2+^ > Mg^2+^ > Na^+^ [41]. Weak cation exchange resins, on the other hand, have more affinity towards monovalent ions. Specifically, the carboxyl group exhibits a very high affinity towards H^+^ which may result in its lower affinity for other ions [42]. Alternatively, the sulfonic acid group has a higher affinity for Ca^2+^ and Mg^2+^ and a low affinity for Na^+^ and H^+^ [42].

A study by Zhang and Chen used EDI to separate ions in groundwater using Amberlite resins with sulfonic acid functional groups and their data indicated that there was no significant preference for divalent over monovalent ions [43]. However, it is important to note that they used different resins, had more types of ions present, and their system was at a much lower ion concentration. Another study using WE-EDI to remove ions from fracking water found that sulfonic acid resins (Amberlite 120 Na^+^) tended to have a preference for divalent cations more than carboxylic acid resins (Dowex MAC 3 H^+^) [44].

### 3.3. FTIR-ATR Spectroscopy Analysis

**Figure 5:**
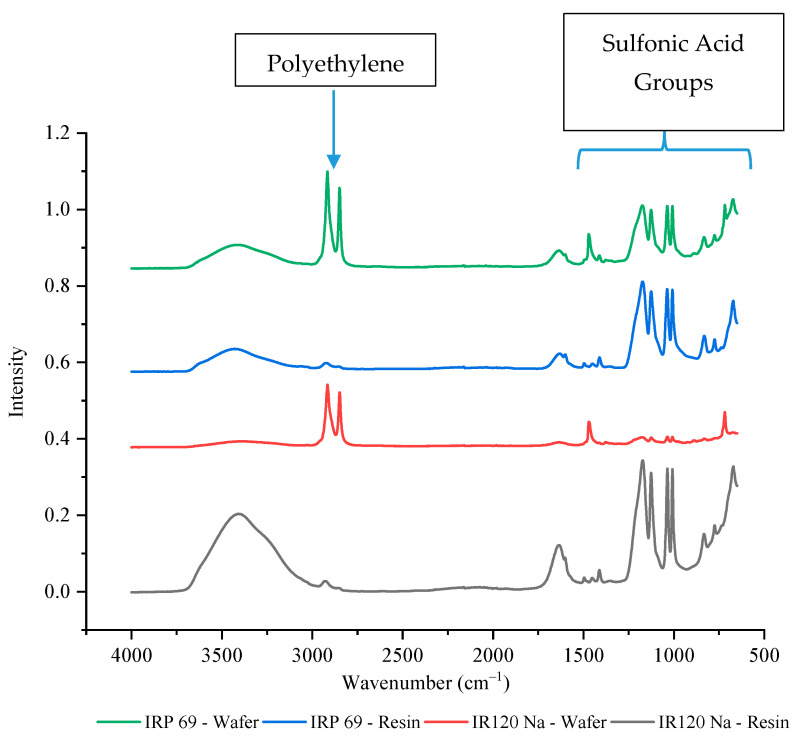
The FTIR-ATR spectrum of strong cation exchange resins and wafers including these resins.

The IR 120 Na^+^ and IRP 69 resins have the same functional group of sulfonic acid which makes the resins strong cation exchangers. Since both resins had the same functional group, it was expected that their current efficiencies and selectivity values would be similar. However, it was observed that the IRP 69 wafer had a current efficiency that was consistently around 100% whereas IR 120 Na^+^ had a lower average value as well as a lot of variability, which made it less desirable for the selective removal of ions. Since these resins have the same chemistry, perhaps the difference in their performance was due to a variation in the accessibility of the active site. To better understand their differences, FTIR-ATR was performed. As shown in Figure 5, four peaks were observed between 1000 cm^−1^ and 1200 cm^−1^ that correspond to sulfonic acid functional groups. The peaks between 1030 to 1200 cm^−1^ have been previously reported to correspond to the symmetric and asymmetric stretching vibration of the −SO^3−^ group of sulfonic acid [45]. The peaks at ~1000 cm^−1^ have been typically associated with an S-O stretch. While these groups were clearly present in IRP 69 wafer, their intensity was much lower in IR 120 Na^+^ wafer which indicated a significant decrease of the sulfur content and exposure of −SO^3−^ groups. Specifically, in the IR 120 Na^+^ wafer, the intensity of the sulfonic acid peaks was around 10% of the resin’s value while for IRP 69 wafer the peaks were 65–70% of the resin’s value (exact values are provided in Supplementary Table S1). This could indicate that polyethylene is covering the IR 120 Na^+^ resin’s larger bead form and thereby decreasing the availability of the sulfonic acid functional groups. This may explain the high variability seen in the current efficiency and selectivity of the IR 120 Na^+^ wafer. 

**Figure 6:**
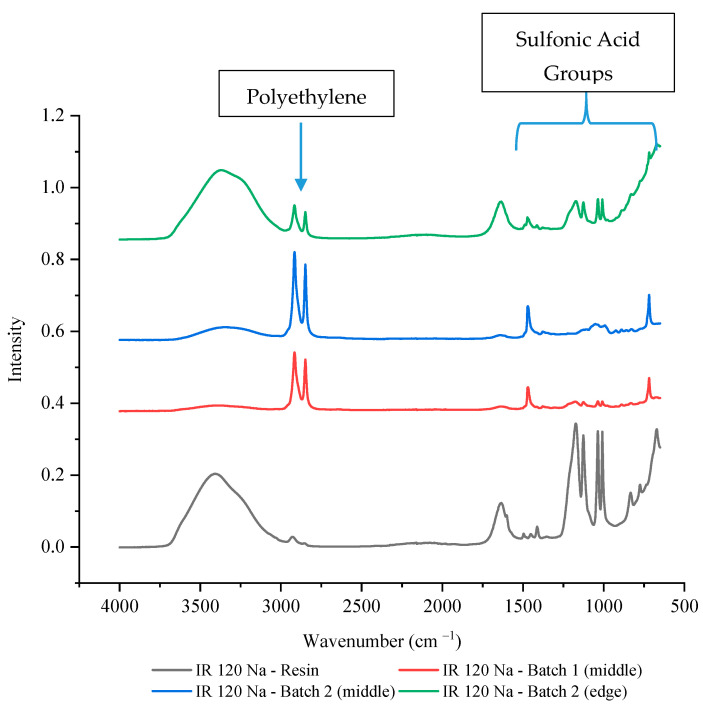
The FTIR-ATR spectrum of IR 120 Na^+^ resin alone and in two different wafers.

To verify that this was not the result of a single batch issue or due to analysis placement, another batch of IR 120 Na^+^ wafer was made and multiple locations were tested using FTIR-ATR. Figure 6 shows that the second batch of IR 120 Na^+^ wafer also had lower intensities of sulfonic acid functional groups compared to the IR 120 Na^+^ resin, especially in the middle of the wafer (~10% of the resin’s value). While the edge of the wafer showed decreased intensity of the sulfonic acid functional groups compared to the resin, it was higher than the middle of the wafer with a value that was between 30–35% of the resin’s value (see Supplementary Table S1 for exact values). This could be due to the resin bead being more exposed at the edge of the wafer than it can be in the middle of the wafer. This finding supports the theory that the availability of the sulfonic acid functional groups of IR 120 Na^+^ have decreased availability possibility due to being covered by the polyethylene binding polymer. 

A recent study by Palakkal et al. using SEM observed that polyethylene was partially covering their cation exchange resin (Purolite PFC100E) which had sulfonic acid functional groups and was a similar size to the Amberlite IR 120 Na^+^ resins at around 0.3 to 0.5 mm [28]. When they used an ionomer binder rather than polyethylene, they observed significantly less coverage of their cation exchange resin. Another possible reason for the difference between the intensity of the sulfonic acid functional groups between the resin and wafer could be due to thermal degradation during the wafer making process. However, a study by Singare et al. showed that during FTIR analysis the sulfonic acid group peaks for Amberlite 120 were present at a significant intensity up to 200 °C (392 °F) while they disappear at around 400 °C (752 °F) [46]. This is well above the wafer making temperature of 250 °F, which further supports the idea that the reduction is due to interactions with the binding polymer. 

**Figure 7:**
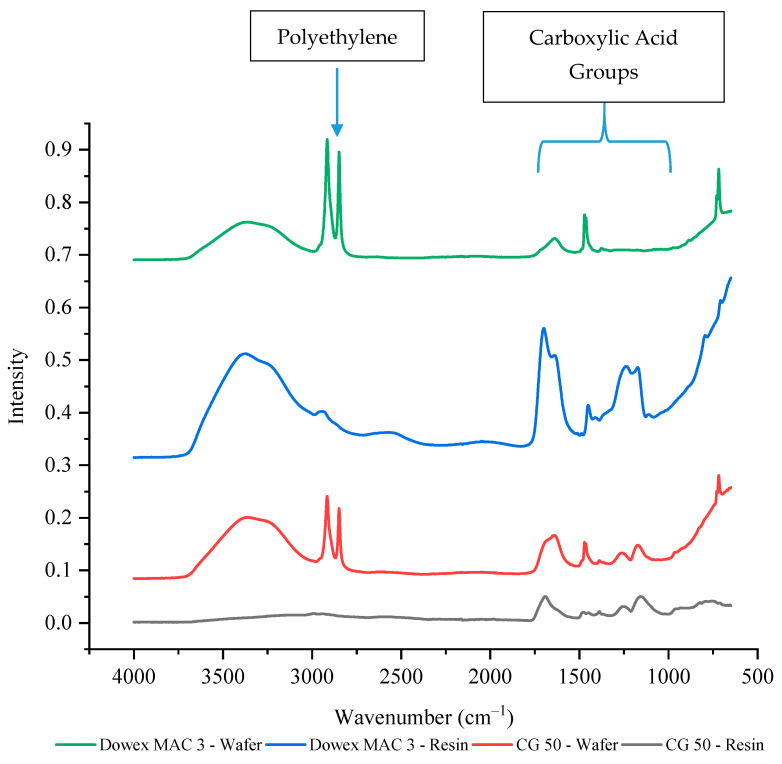
The FTIR-ATR spectrum of weak cation exchange resins and wafers formed using these resins.

The weak cation exchange resins both have carboxylic acid functional groups which should have a peak between 1760 to 1690 cm^−1^ for the C=O stretch and a peak between 1320 to 1210 cm^−1^ for the C–O stretch [47]. Unlike the strong cation exchange resin wafers, the current efficiencies and selectivity values were similar between the two weak cation exchange resin wafers. However, the size of the cation exchange resins was also different between the Dowex MAC 3 H^+^ (bead form) and the CG 50 (powder form). As shown in Figure 7, the intensity of the carboxylic acid functional groups for powdered CG 50 resin was only about 20% of the intensity of the Dowex MAC 3 H^+^ bead resin. Once incorporated into a wafer, the Dowex MAC 3 H^+^ wafer had around 10% of the peak intensity of the resin alone (exact values are provided in Supplementary Table S2). For the CG 50 (powder) wafer, the wafer peak intensities were actually around 40–50% higher than the resin alone. As the CG 50 resin intensities were so much lower than the Dowex MAC 3 H^+^, it is possible that interference from other groups present in the wafer (from the polyethylene or anion exchange resin) led to the higher intensities. 

**Figure 8:**
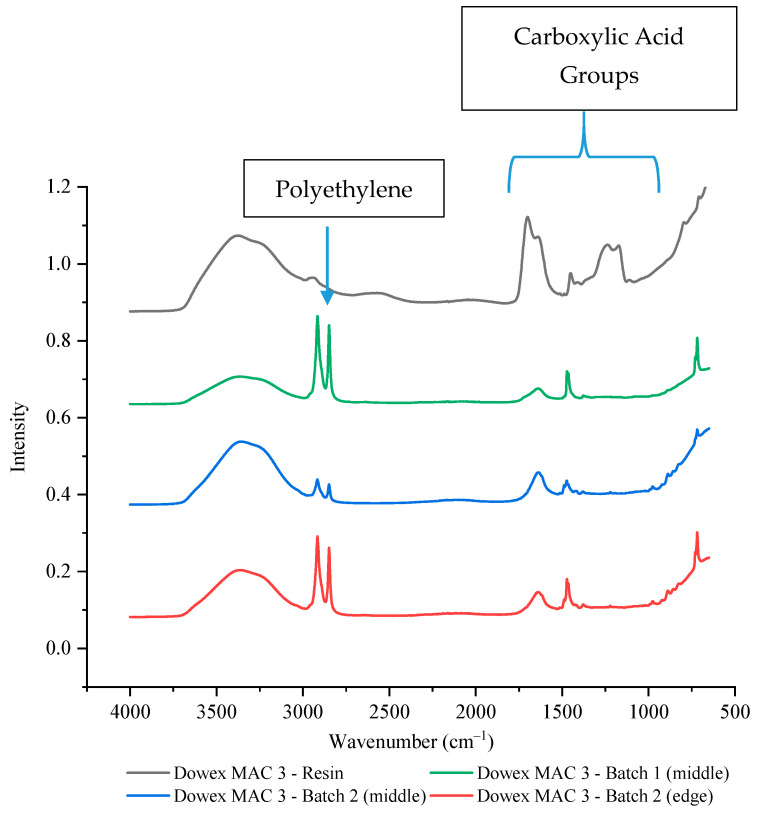
The FTIR-ATR spectrum of Dowex MAC 3 H^+^ resin alone and in two different wafers.

To confirm that the bead resins led to less availability of the function groups, two different batches and multiple wafer positions of Dowex MAC 3 H^+^ resin wafers were tested by FTIR-ATR. In both batches, the intensity of the carboxylic acid functional groups was significantly reduced at both the edge and the middle with intensity values of around 10–18% of the resin alone (Figure 8, Supplementary Table S2). It is interesting to note that this reduction did not appear to have any effect on the performance of the Dowex MAC 3 H^+^ resin wafer unlike what was observed with the strong cation exchange resin bead (IR 120 Na^+^).

The difference might be explained by how the functional groups interact with the polyethylene. Sulfonic acid functional groups tend to attach to polyethylene. This behavior can be positive for membrane processes as it has been reported to increase ion transport [48] and lower fouling [49]. However, this attachment may be decreasing the availability of sulfonic acid functional groups in the wafer and thereby, decreasing the efficiency and the performance of the resin wafer for the removal of ions from high concentration wastewaters. 

### 3.4. Performance Comparison of the Powdered and Bead Form IR 120 Na^+^

The interaction of polyethylene with the sulfonic acid groups does not fully explain the difference in performance between the two strong cation exchange resins. Therefore, we decided to evaluate if decreasing the particle size of the IR 120 Na^+^ resin would increase its performance when incorporated in a wafer. Using the same method outlined in Section 2.3, a new batch of wafers were produced from ground IR 120 Na^+^ resins.

**Figure 9:**
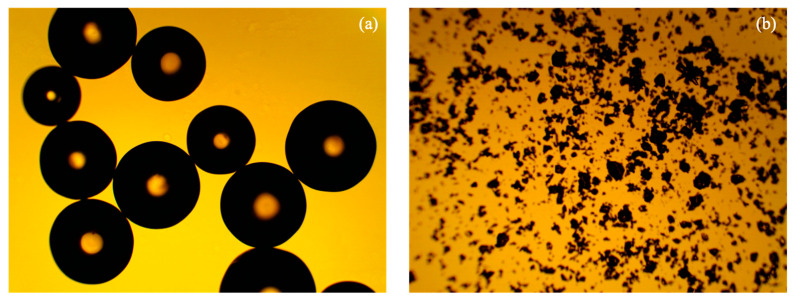
Optical microscopy images of (**a**) unground IR 120 Na^+^ resin and (**b**) ground IR 120 Na^+^ resin.

Figure 9 clearly shows the particle size difference between the original IR 120 Na^+^ resin and the ground IR 120 Na^+^ resin. The original IR 120 Na^+^ resin had a particle diameter of 536 ± 65 µm (N = 8) and the ground IR 120 Na^+^ resin had a particle diameter of 30 ± 20 µm (N = 1101).

**Figure 10:**
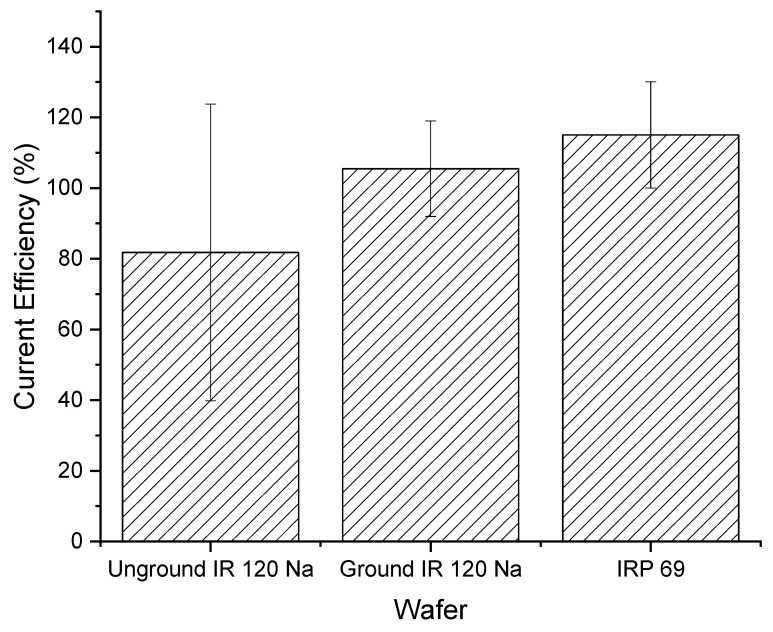
Current efficiencies for unground bead form IR 120 Na^+^ and ground IR 120 Na^+^.

Figure 10 shows the ground IR 120 Na^+^ wafer had a higher and less variable current efficiency compared to the unground IR 120 Na^+^ wafer. In addition, the ground IR 120 Na^+^ wafer looked similar in performance to the powdered IRP 69 resin wafer. However, it is important to note that all the current efficiency values were statistically the same (*p* > 0.4).

**Figure 11:**
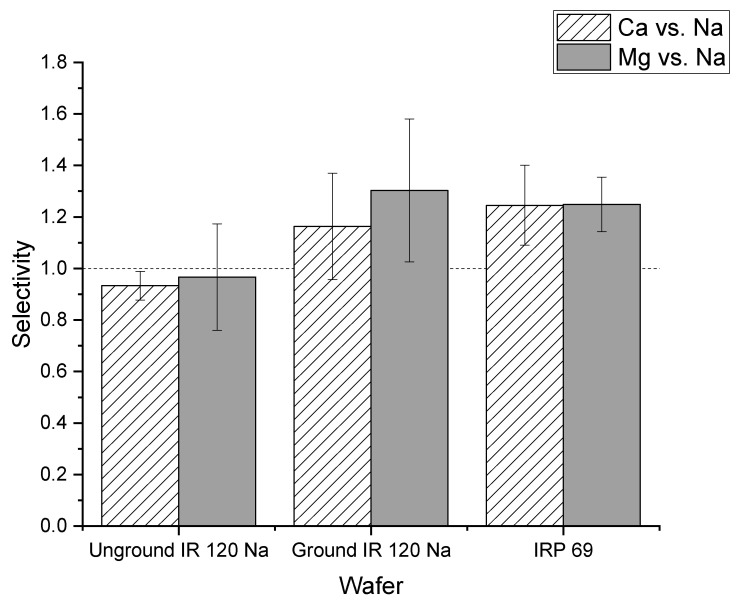
The selectivity of the unground IR 120 Na^+^ and the ground IR 120 Na^+.^

In addition to current efficiency, the cation selectivity of the two different forms of the IR 120 Na^+^ resin in wafers were compared. As shown in Figure 11, the average selectivity of calcium to sodium of ground IR 120 Na^+^ wafer was greater than one which indicated that the ground IR 120 Na^+^ wafer preferentially transported calcium ions over sodium. For the unground IR 120 Na^+^ resin, the selectivity was close to one which indicated that there was not a strong preference for the transport of sodium or calcium ions. However, statistical analysis showed that the difference between the wafer produced from ground versus unground IR 120 Na^+^ for calcium selectivity was not significant (*p* > 0.05). A similar situation was observed for the selectivity of magnesium over sodium. While the ground IR 120 Na^+^ demonstrated selectivity for magnesium over sodium which the unground did not, their values were statistically the same (*p* > 0.1) When compared to the powder resin IRP 69, the selectivity of ground IR 120 Na^+^ resin wafers were statistically the same (*p* > 0.05) for both calcium to sodium and magnesium to sodium. Overall, significantly better performance was produced by wafers composed of the ground IR 120 Na^+^ resin compared to its bead form which indicates the importance of strong cation exchange resin size when being used in an electrodeionization wafer. 

## 4. Conclusions

Four different cation exchange resins were tested for their performance in electrodeionization wafers for the removal of monovalent and divalent cations. Wafers made from weak cation exchange resins and strong cation exchange resins showed similar current efficiencies, although they showed differences in their degree of variability. Based on the selectivity values, weak cation exchange resins seemed to favor the transport of the monovalent ion (sodium), while strong cation exchange resins either had no preference or a preference for the divalent ions (calcium and magnesium), which are usually the more valuable ions in wastewaters. 

In addition, the strong cation exchange resins in powder form generally performed better in wafers for the selective removal of divalent ions. This could be due to a more homogeneous mixing with the other wafer materials or it could be due to differences in how it interacts with the polyethylene binding polymer during the formation of wafers. Specifically, wafers formed from IRP 69 strong cation exchange resin in powder form gave the most promising results for the removal of divalent ions. 

The positive impact of powder form was also verified by testing two different forms (ground vs. unground) of the same strong cation exchange resin for their performance in electrodeionization wafers for the removal of monovalent and divalent ions. The resin in powder form from the grinding process showed higher overall current efficiencies compared to the unground form (bead) of the resin. Based on the selectivity values, the ground resin seemed to favor the transport of divalent ions (calcium and magnesium) that are more valuable, while the unground resin did not show any preference for either monovalent or divalent ions.

## Figures and Tables

**Table 1:** Cation exchange resins and their properties.

Name	Functional Group	Matrix	Particle Size (Mesh) *	Exchange Capacity (eq/L)
Amberlite IR120 Na^+^	Sulfonic Acid	Styrene-divinylbenzene (gel)	16–50 mesh (0.297 to 1.19 mm)	≥2.0
Amberlite IRP 69	Sulfonic Acid	Crosslinked styrene-divinylbenzene	100–200 mesh (0.074 to 0.149 mm)	5
Dowex MAC 3 H^+^	Carboxylic Acid	Polyacrylic-divinylbenzene (gel)	16–50 mesh (0.297 to 1.19 mm)	3.8
Amberlite CG 50	Carboxylic Acid	Methacrylic (macroporous)	100–200 mesh (0.074 to 0.149 mm)	3.5

*: Mesh is a measurement for the particle size that is used to determine the particle size distribution of a granular material. Particle size conversion (mesh to mm) was determined from [38].

## Data Availability

Data is contained within the article or supplementary material.

## Supplementary Materials

The following are available online at https://www.mdpi.com/2077-0375/11/1/45/s1, Table S1: FTIR sulfonic acid functional group peak intensity values for strong cation exchange resins alone and incorporated into wafers, Table S2: FTIR carboxylic acid functional group peak intensity values for weak cation exchange resins alone and incorporated into wafers.
