# Supplementary material for: Effects of Resin Chemistries on the Selective Removal of Industrially Relevant Metal Ions Using Wafer-Enhanced Electrodeionization
Source: Membranes (Basel). 2021 Jan 9;11(1):45. doi: 10.3390/membranes11010045 (PMC7827004; doi:10.3390/membranes11010045)
Supplement: Supplementary file 1 [file membranes-11-00045-s001.pdf]

Supplementary Materials: Effects of Resin Chemistries on the Selective Removal of Industrially Relevant Metal Ions Using Wafer-Enhanced Electrodeionization

Humeyra B. Ulusoy Erol, Christa N. Hestekin, and Jamie A. Hestekin *

Supplementary Table S1: FTIR sulfonic acid functional group peak intensity values for strong cation exchange resins alone and incorporated into wafers

Wavelength (cm^{-1})	IR 120 Na^+ resin	IR 120 Na^+ wafer B1 mid	IR 120 Na^+ wafer B2 mid	IR 120 Na^+ wafer B2 edge	IRP 69 resin	IRP 69 wafer
1173	0.3435	0.0254	0.0181	0.1062	0.2431	0.1643
1126	0.3110	0.0243	0.0288	0.1032	0.2172	0.1531
1036	0.3222	0.0266	0.0430	0.1118	0.2221	0.1633
1008	0.3205	0.0263	0.0379	0.1112	0.2218	0.1629

B1 = batch 1, B2 = batch 2, mid = middle of wafer

Supplementary Table S2: FTIR carboxylic acid functional group peak intensity values for weak cation exchange resins alone and incorporated into wafers

Wavelength (cm^{-1})	Dowex MAC 3 H^+ resin	Dowex MAC 3 H^+ wafer B1 mid	Dowex MAC 3 H^+ wafer B2 mid	Dowex MAC 3 H^+ wafer B2 edge	CG 50 resin	CG 50 wafer
1760 - 1690	0.2463	0.0260	0.0406	0.0340	0.0504	0.0713
1320 - 1210	0.1742	0.0198	0.0314	0.0287	0.0315	0.0496

B1 = batch 1, B2 = batch 2, mid = middle of wafer
